# Efficacy of antifibrotic drugs, nintedanib and pirfenidone, in treatment of progressive pulmonary fibrosis in both idiopathic pulmonary fibrosis (IPF) and non-IPF: a systematic review and meta-analysis

**DOI:** 10.1186/s12890-021-01783-1

**Published:** 2021-12-11

**Authors:** James Patrick Finnerty, Aravind Ponnuswamy, Prosjenjit Dutta, Ammar Abdelaziz, Hafiz Kamil

**Affiliations:** 1 Department of Respiratory Medicine, Countess of Chester Hospital NHS Trust, Liverpool Road, Chester, CH2 1UL UK; 2 University of Chester Medical School, Chester, UK; 3 The Walton Centre, Liverpool, UK

**Keywords:** Idiopathic pulmonary fibrosis, Nintedanib, Pirfenidone

## Abstract

**Background:**

*Research questions* To compare the efficacy of nintedanib and pirfenidone in the treatment of progressive pulmonary fibrosis; and to compare the efficacy of anti-fibrotic therapy (grouping nintedanib and pirfenidone together) in patients with IPF versus patients with progressive lung fibrosis not classified as IPF.

**Study design and methods:**

A search of databases including MEDLINE, EMBASE, PubMed, and clinicaltrials.gov was conducted. Studies were included if they were randomised controlled trials of pirfenidone or nintedanib in adult patients with IPF or non-IPF patients, and with extractable data on mortality or decline in forced vital capacity (FVC). Random effects meta-analyses were performed on changes in FVC and where possible on mortality in the selected studies.

**Results:**

13 trials of antifibrotic therapy were pooled in a meta-analysis (with pirfenidone and nintedanib considered together as anti-fibrotic therapy). The change in FVC was expressed as a standardised difference to allow pooling of percentage and absolute changes. The mean effect size in the IPF studies was − 0.305 (SE 0.043) (*p* < 0.001) and in the non-IPF studies the figures were − 0.307 (SE 0.063) (*p* < 0.001). There was no evidence of any difference between the two groups for standardised rate of FVC decline (*p* = 0.979). Pooling IPF and non-IPF showed a significant reduction in mortality, with mean risk ratio of 07.01 in favour of antifibrotic therapy (*p* = 0.008). A separate analysis restricted to non-IPF did not show a significant reduction in mortality (risk ratio 0.908 (0.547 to 1.508), *p* = 0.71.

**Interpretation:**

Anti-fibrotic therapy offers protection against the rate of decline in FVC in progressive lung fibrosis, with similar efficacy shown between the two anti-fibrotic agents currently in clinical use. There was no significant difference in efficacy of antifibrotic therapy whether the underlying condition was IPF or non-IPF with progressive fibrosis, supporting the hypothesis of a common pathogenesis. The data in this analysis was insufficient to be confident about a reduction in mortality in non-IPF with anti-fibrotic therapy.

*Trial Registration* PROSPERO, registration number CRD42021266046.

## Introduction

Idiopathic pulmonary fibrosis (IPF) is the most common type of idiopathic interstitial pneumonia (IIP). IIPs are spontaneously occurring (idiopathic) diffuse parenchymal lung diseases. IPF is defined as a spontaneously occurring (idiopathic) specific form of chronic fibrosing interstitial pneumonia limited to the lung and associated with a pattern of Usual Interstitial Pneumonia (UIP) on imaging or histology [1]. NICE guidelines for the diagnosis of Interstitial Lung Disease (ILD), prior to consideration for anti-fibrotic therapy, stipulate that the diagnosis of ILD has been made by a multidisciplinary team (MDT) [2].

Many cases of lung fibrosis, including progressive disease, are not UIP, either because they belong to another clearly defined group, or because they are unclassifiable despite MDT analysis. The other IIPs include nonspecific interstitial pneumonia (NSIP), desquamative interstitial pneumonia (DIP), respiratory bronchiolitis associated interstitial lung disease (RB-ILD), acute interstitial pneumonia (AIP), lymphocytic interstitial pneumonia (LIP), and cryptogenic organizing pneumonia (COP). All of these, plus hypersensitivity pneumonitis, may be treated differently from IPF initially, but can lead to progressive and ultimately fatal fibrosis; and anti-fibrotic therapy with nintedanib or pirfenidone is not licensed in the UK in these circumstances.

Two anti-fibrotic drugs are currently recommended (under specified circumstances) as treatment options in ILD in the UK: pirfenidone [3] and nintedanib [4]. Their modes of action are not completely understood but there is evidence of considerable overlap [5, 6], and they have similar effects in clinical practice. For nintedanib, the evidence considered came from 3 multicentre trials, and showed statistically significant reduction in the rate of decline in Forced Vital Capacity (FVC), and a non-significantly reduced mortality. For pirfenidone, the evidence came from 4 randomised trials: there was a statistically significant difference in rate of decline in FVC as percentage predicted in favour of pirfenidone. All the above studies were in populations of patients with confirmed ILD of IPF type. There is now more recent evidence of use of these anti-fibrotic drugs in lung fibrosis that is not classified as IPF [7–9].

Our two central aims were:

1. To assess efficacy comparing nintedanib and pirfenidone in the treatment of progressive pulmonary fibrosis (grouping IPF and non-IPF together), using changes in FVC and mortality relative to controls as outcomes.
2. For current anti-fibrotic therapy (grouping nintedanib and pirfenidone together), to assess any difference in efficacy between IPF and progressive lung fibrosis not classified as IPF, using changes in FVC and mortality relative to controls as outcomes.

Our primary analysis was on changes in FVC, with a secondary analysis based on total deaths during studies.

## Methods

We performed a systematic review and meta-analysis. It was performed in accordance with the Preferred Reporting Items for Systematic Reviews and Meta-analyses (PRISMA) statement. The trial was registered with PROSPERO: registration number CRD42021266046.

### Search strategy and study selection

A search of databases using Healthcare Databases for the NHS in England (HDAS) was performed. The searches were from 1990 (since no trials in human subjects using pirfenidone or nintedanib predate that time) until the end of July 2021. The databases searched were MEDLINE, EMBASE and PubMed. The precise search strategy from these databases is given below:

MEDLINE: 1,611 combined results for *37*: ~ "(((pirfenidone).ti,ab OR (Esbriet).ti,ab OR (nintedanib).ti,ab OR (Ofev).ti,ab OR (Vargatef).ti,ab OR (antifibrotic).ti,ab OR (anti-fibrotic).ti,ab OR (anti fibrotic).ti,ab) AND ((lung fibrosis).ti,ab OR (pulmonary fibrosis).ti,ab OR (idiopathic pulmonary fibrosis).ti,ab OR (IPF).ti,ab OR (INTERSTITIAL LUNG*).ti,ab OR (UIP).ti,ab OR (NSIP).ti,ab)) [DT 2001–2021] [Languages English] [Humans]".

PUBMED: 2,456 combined results for *(53 AND 54)*: ~ "((pirfenidone).ti,ab OR (Esbriet).ti,ab OR (nintedanib).ti,ab OR (Ofev).ti,ab OR (Vargatef).ti,ab OR (antifibrotic).ti,ab OR (anti-fibrotic).ti,ab OR (anti fibrotic).ti,ab) AND ((lung fibrosis).ti,ab OR (pulmonary fibrosis).ti,ab OR (idiopathic pulmonary fibrosis).ti,ab OR (IPF).ti,ab OR (INTERSTITIAL LUNG*).ti,ab OR (UIP).ti,ab OR (NSIP).ti,ab).

EMBASE: 1,666 combined results for *18*: ~ "(((pirfenidone).ti,ab OR (Esbriet).ti,ab OR (nintedanib).ti,ab OR (Ofev).ti,ab OR (Vargatef).ti,ab OR (antifibrotic).ti,ab OR (anti-fibrotic).ti,ab OR (anti fibrotic).ti,ab) AND ((lung fibrosis).ti,ab OR (pulmonary fibrosis).ti,ab OR (idiopathic pulmonary fibrosis).ti,ab OR (IPF).ti,ab OR (INTERSTITIAL LUNG*).ti,ab OR (UIP).ti,ab OR (NSIP).ti,ab)) [DT 2001–2021] [English language] [Languages English] [Human age groups Adult 18 to 64 years OR Aged 65 + years] [Humans]".

By inspection of titles, papers were saved from each search, and HDAS was used to remove duplicates. The remaining papers were then screened by their abstracts as outlined below.

A search of ClinicalTrials.gov was performed: the conditions used were idiopathic pulmonary fibrosis and pulmonary fibrosis; the limits were Adults (any age); and the interventions were nintedanib and pirfenidone and BIBF 1120. Data from ClinicalTrials.gov was cross-tabulated with the above search, and used to check data extracted for use in the meta-analysis. A search of PROSPERO was performed for meta-analyses including interstitial lung disease as a subject: no analyses were completed or proposed that are current and cover the same ground as the current study.

### Data inclusion and exclusion

Abstracts of above searches were reviewed by two investigators independently, looking for possible studies for inclusion. The criteria were: 1. the study should include reference to use of pirfenidone or nintedanib, in lung disease in adult subjects; and 2. the abstract should report the results of a trial. Full text papers fulfilling the above criteria were obtained. Studies were excluded if a full text published in the English language was not available, and if studies were in animal or laboratory models.

### Data extraction

Data were independently extracted by two investigators (AP and JF). The study design, antifibrotic type, study duration, number of subjects, changes in FVC (however recorded), and all-cause mortality during the study period were tabulated. Disagreements were settled by a third party (PD).

### Quality assessment

Risk of bias assessment was evaluated using the Cochrane risk-of-bias tool for randomised controlled trials, by two investigators (AP and JF), and disagreements settled by a third party (PD).

### Statistical analysis

A meta-analysis was performed on two separate variables: change in FVC (expressed as standardised change to account for different metrics in the papers) and all-cause mortality, treated as a binomial variable. Data were pooled using random effects models. Sensitivity studies were performed using one study removed. Heterogeneity was assessed using the Q test, with a *p* value of < 0.10 regarded as significant. If heterogeneity was demonstrated, an estimate of tau and its 95% confidence intervals was made. Publication bias was assessed by inspection of funnel plots. All analyses were performed using the software Comprehensive Meta-Analysis [10].

## Results

**Fig. 1:**
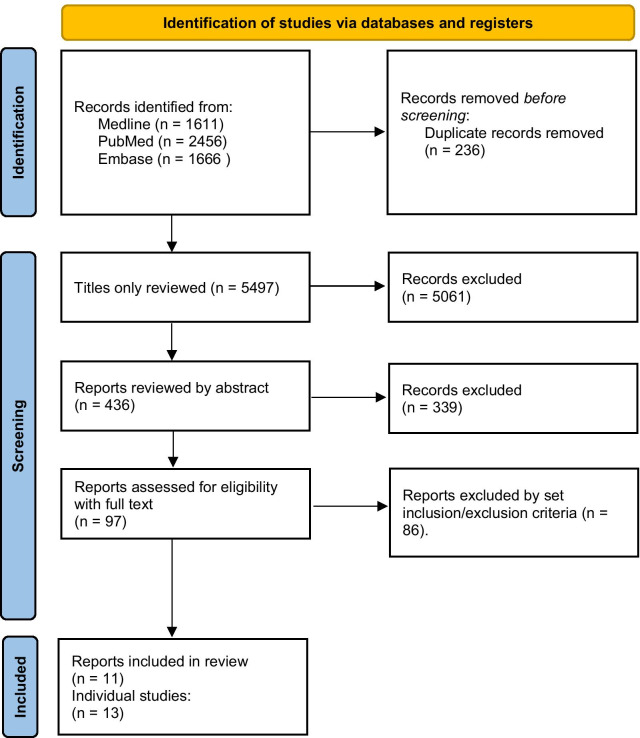
The PRISMA flow diagram for systematic reviews

The number of studies initially identified by the search are given in Fig. 1. Ninety-seven papers including relevant trials underwent full-text review, of which 11 papers covering 13 trials were included in the final analysis (Fig. 1). The studies are referenced here [7–9, 11–18] and are tabulated in Table 1. In the event, no disagreement over included studies occurred. The types of non-IPF entered into studies are summarised in Table 2.

**Table 1:** Summary of included studies

Studies	Year published	Study period (months)	Medication	Disease	N in drug group	N in placebo group	Metric of FVC change used in meta-analysis	All-cause mortality available
Richeldi et al. TOMORROW trial	2011	12	Nintedanib	IPF	84	83	mls/year	Yes
Richeldi et al. INPULSIS trial 1	2014	12	Nintedanib	IPF	309	204	mls/year	Yes
Richeldi et al. INPULSIS trial 2	2014	12	Nintedanib	IPF	329	219	mls/year	Yes
Flaherty et al. INBUILD trial	2019	12	Nintedanib	Non-IPF	206	206	mls/year	Yes
Distler et al. NCT 02597933	2019	12	Nintedanib	Non-IPF	288	288	mls/year	Yes
Azuma et al.	2005	9	Pirfenidone	IPF	73	36	mls over 9 months	No
Taniguchi et al.	2010	12	Pirfenidone	IPF	108	104	mls/year	No
Noble et al. CAPACITY NCT 00287729	2011	18	Pirfenidone	IPF	174	174	% predicted FVC	Yes
Noble et al. CAPACITY NCT 00287716	2011	18	Pirfenidone	IPF	171	173	% predicted FVC	Yes
Huang et al.	2015	11	Pirfenidone	IPF	38	38	mls over study period	Yes
King et al. ASCEND trial	2014	12	Pirfenidone	IPF	278	277	Slope of fall in FVC mls	Yes
Maher et al.	2020	6	Pirfenidone	Non-IPF	118	119	mls FVC over study period	Yes
Behr et al.s	2021	11	Pirfenidone	Non-IPF	35	32	% predicted FVC	Yes

**Table 2:** Diseases included in non-IPF studies

Studies	Year published	Medication	Diseases Included
Flaherty et al. INBUILD trial	2019	Nintedanib	iNSIP^1^, uIIP^2^, HP^3^, RA-ILD^4^, MCTD-ILD^5^, SSc-ILD^6^ and others
Distler et al. NCT 02597933	2019	Nintedanib	SSc-ILD^6^
Maher et al.	2020	Pirfenidone	Unclassifiable ILD
Behr et al.	2021	Pirfenidone	iNSIP^1^, HP^3^, RA-ILD^4^, MCTD-ILD^5^, SSc-ILD^6^, asbestos-related fibrosis

Key to abbreviations for Table 2:

1. Idiopathic non-specific interstitial pneumonitis; 2. unclassifiable idiopathic interstitial pneumonitis; 3. Chronic hypersensitivity pneumonitis; 4. Rheumatoid arthritis related interstitial lung disease; 5. Mixed connective tissue disease associated ILD; 6. Systemic sclerosis-associated ILD.

Of the 13 trials included, all had data extracted on FVC changes, and 11 had data available on all-cause mortality (this was pooled for the two INPULSIS trials and the two CAPACITY trials, so the total of separate trials analysed for all-cause mortality reduced to nine). The main variables of these studies are summarised in Table 1. All were randomised controlled trials.

### Bias assessment

A consensus on bias assessment was achieved. Three studies were identified as having “some concerns” in one domain of five. The study of Maher et al. (2020) was rated as “some concerns” on domain 5 (selection of the reported result): this was because the primary outcome of home spirometry could not be used because of some impossible or implausible values, and the site spirometry (which is used in all the studies) is presented in this analysis instead. In Behr et al. (2021), the fact that a large quantity of data had to be imputed led to “some concerns” in domain 3 (missing outcome data: the algorithm gave the risk of bias as low). In Azuma et al. (2005), we assessed as having “some concerns” in domain 5 (selection of reported result), as the FVC data used was among several outcomes reported, as the primary outcome (Sp02 fall during 6MWT) had not proved significant. All other studies were rated as low risk of bias on all domains.

### Qualitative review of studies

In several of the trials, co-administration of medication previously thought to have efficacy in IPF was noted. In the study by Huang et al. [17], all patients received N-acetyl cysteine (NAC) 600 mg three times daily. Evidence from an adequately powered RCT showed no effect of NAC on rate of FVC decline or mortality in IPF, so it is unlikely to have altered or biased the efficacy of anti-fibrotic therapy [19].

In the TOMORROW and INPULSIS trials, up to 15 mg daily of prednisolone was permitted: its use was well balanced between the treatment and control groups in all those trials, with about 21% of the study population on prednisolone therapy. In the INPULSIS trials, after 6 months, treating physicians could increase the dose of prednisolone, or add azathioprine, ciclosporin or cyclophosphamide: the numbers in which this happened are not given in the paper or its supplement.

In the study by Azuma et al., azathioprine was not permitted, but prednisone up to a dose of 10 mg per day was permitted: the frequency of oral corticosteroid use was not stated in the paper, but it is clarified that acute exacerbations were treated with high dose corticosteroids. In the study by Taniguchi et al., the same entry criteria were used, and this time the paper states that 8% of the pirfenidone group and 5% of the control group were on oral corticosteroids. In the CAPACITY trials, “other IPF therapy” was excluded. In Huang et al., prednisone up to 15 mg/day was permitted, and was a past or current medication, possibly also with azathioprine, in 74% of the pirfenidone group and 68% of the control group. In the ASCEND trial, no patients were receiving azathioprine, and prednisone was only permitted if required for conditions other than IPF: the number to whom that applied is not stated, but it is reasonable to suppose it was very low. In summary, of the studies of nintedanib in IPF, about 21% of the study population was on prednisolone up to 15 mg daily. Of the studies of pirfenidone in IPF, the majority of subjects were in the CAPACITY and ASCEND trials, where prednisolone was not permitted, so any effect of anti-inflammatory therapy is likely to be nugatory.

The above studies were in IPF. Those below are the four studies in non-IPF, where anti-inflammatory therapy was administered to some patients in all the studies reviewed. In the study by Maher et al., subjects were stated to be not on oral corticosteroids or azathioprine, and 5% were on mycophenolate in both pirfenidone and control groups. In the study by Behr et al., for the pirfenidone group 27% were on monotherapy with oral corticosteroids, 36% were on combination therapy with oral corticosteroids and another agent, usually azathioprine (17% of the treatment group) or mycophenolate, with similar figures for the control group. In the INBUILD study, up to 20 mg prednisolone was permitted: at baseline, 17.2% of subjects on nintedanib and 17.8% of control subjects were on at least one anti-inflammatory drug, mainly non-biologics such as methotrexate, with less than 1% on prednisolone and 0% on azathioprine. Over the course of the study period, anti-inflammatory therapy was added or altered in 10.8% of the nintedanib group and 21.1% of the control group (mainly oral corticosteroids). In the Distler study of patients with Systemic Sclerosis, 48.3% of the nintedanib group were on mycophenolate, and 8% were on methotrexate, with similar figures for both drugs in the placebo group. Patients who had recently received oral corticosteroids or azathioprine were excluded.

### Effects of antifibrotics on standardised changes in FVC for IPF and non-IPF considered together

**Fig. 2:**
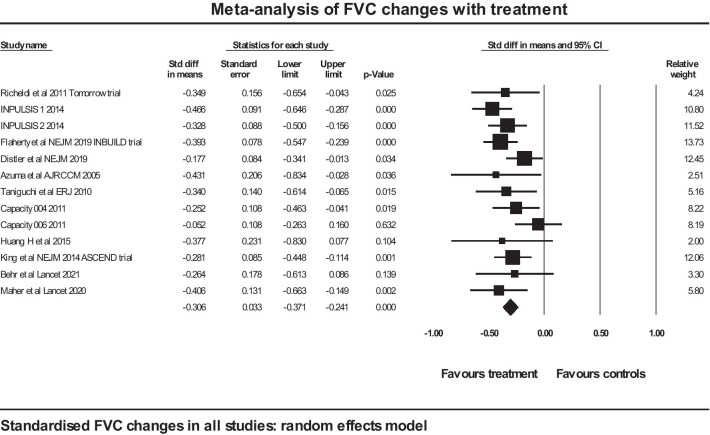
Forest plot of meta-analysis: FVC changes in all studies

A meta-analysis was performed on included trials assessing the effect of antifibrotic therapy on the rate of decline in FVC, using a standardised effect size to allow comparison of studies using different metrics for the rate of decline in FVC. The total number of subjects in trials in IPF was 2872 (of whom 1564 took the active medication), and the total number of subjects in trials in non-IPF was 1292, of whom 647 took active medication. Of the studies in IPF, 3 were with nintedanib and 6 were with pirfenidone. Of the studies in non-IPF, 2 were with nintedanib and 2 were with pirfenidone (Table 1). The forest plot is shown in Fig. 2.

**Fig. 3:**
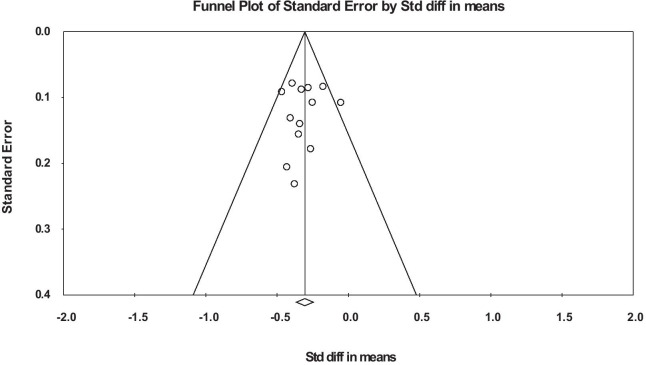
Funnel plot of studies analysed by standardised difference in means between treatment and controls

The overall mean effect size is − 0.306 (SE 0.033), *p* < 0.001. The Cochrane Q is 13.9, df 12, *p* = 0.306, so no statistically significant heterogeneity was shown. A sensitivity analysis for the mean effect size was performed using one study removed and re-analysis: the mean effect size showed little variation, with a range of − 0.286 to − 0.328. A funnel plot of the studies did not show any evidence of publication bias (Fig. 3).

**Fig. 4:**
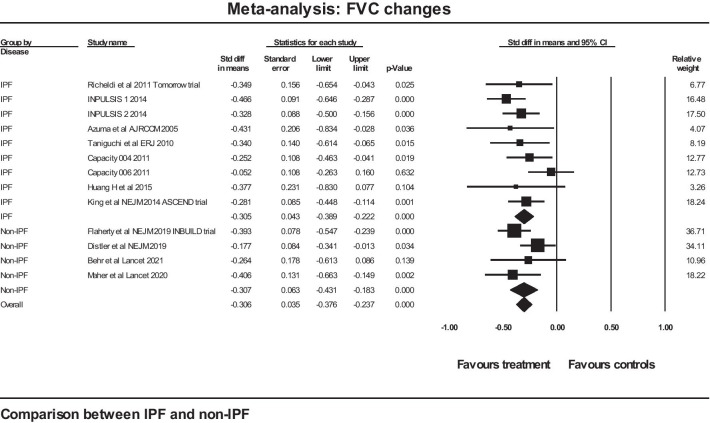
Comparison of two groups of studies: IPF versus non-IPF for standardised FVC change in response to therapy

A further analysis was performed between two groups of studies (those on IPF and those on non-IPF) using a mixed effects model with the difference between the two groups treated as a fixed effect. The forest plot for that analysis is shown in Fig. 4. The mean effect size in the IPF studies was − 0.305 (SE 0.043) (*p* < 0.001) and in the non-IPF studies the figures were − 0.307 (SE 0.063) (*p* < 0.001). The Q statistic for the group difference was 0.001, df 1, with *p* = 0.979: there was no evidence of any difference between the two groups for standardised rate of FVC decline.

Although when all studies are considered together, no significant heterogeneity was shown, we can analyse the data as if there were heterogeneity, and calculate a prediction interval for the distribution of true effects: this gives an interval for the true effect size (disregarding sampling error) which would be found in 95% of studies from a comparable universe of similar studies, using either pirfenidone or nintedanib, and performed in patients with IPF or non-IPF. That interval, expressed as a standardised difference between treatment and control groups is − 0.18 to − 0.43.

### Effects of nintedanib and pirfenidone analysed separately on FVC change for IPF and non-IPF considered together

**Fig. 5:**
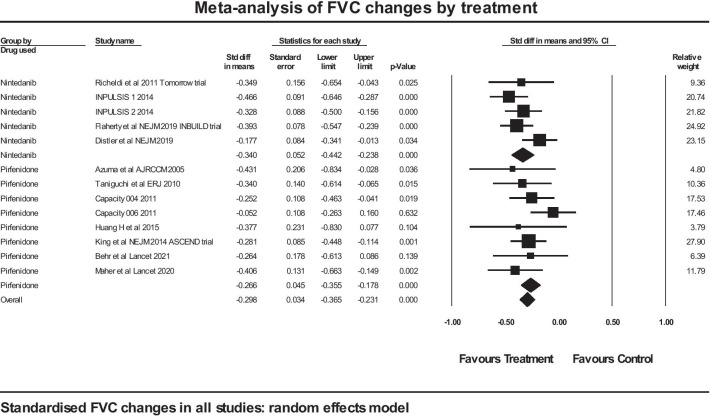
Comparison of nintedanib and pirfenidone on standardised changes in FVC

A further analysis was performed between two groups of studies, namely those where nintedanib was the treatment and those where pirfenidone was the treatment. This was performed over all studies in the meta-analysis, so both IPF and non-IPF. Again, this used a mixed effects model with the difference between the two groups treated as a fixed effect. The forest plot for that analysis is shown in Fig. 5. The mean effect size in the nintedanib studies was − 0.340 (SE 0.052) (*p* < 0.001) and in the pirfenidone studies the figures were − 0.266 (SE 0.045) (*p* < 0.001). The Q statistic for the group difference was 1.139, df 1, with *p* = 0.286: there was no statistically significant evidence of any difference between the two groups by anti-fibrotic treatment given.

### Effects of antifibrotics on all-cause mortality in patients with IPF and non-IPF considered together

**Fig. 6:**
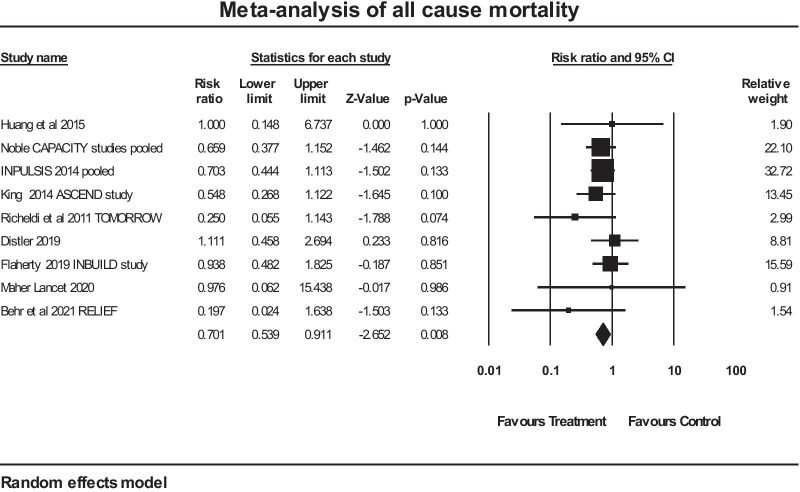
Meta-analysis of all-cause mortality

A meta-analysis of 9 studies where all-cause mortality was stated in the publications used in this paper was performed. The forest plot of that analysis is given in Fig. 6. The overall mean risk ratio for all-cause mortality was 0.701 in favour of anti-fibrotic therapy (confidence intervals 0.539 to 0.911), *p* = 0.008. The Q value was 5.613, df 8, *p* = 0.69, so no evidence of heterogeneity was evident. A further analysis was performed grouping the studies by disease type: IPF (n = 5) versus non-IPF (n = 4). The estimate for risk ratio (95% interval) for the IPF studies was 0.637 (0.469 to 0.866), *p* = 0.004. The estimate for risk ratio (95% interval) for the non-IPF studies was 0.908 (0.547 to 1.508), *p* = 0.71. An all-cause mortality benefit in the non-IPF group was not demonstrated.

## Discussion

This systematic review and meta-analysis showed that, when IPF and non-IPF studies are grouped together, there is an overall reduction in all-cause mortality (pooled risk ratio of 0.701, CI 0.539 to 0.911), and a clear-cut effect in reducing the rate of decline in FVC (pooled estimate of standardised difference from placebo − 0.306, SE 0.033) attributable to anti-fibrotic therapy. While a pooled effect on mortality was not demonstrated in the non-IPF studies in isolation, the beneficial effect on decline in FVC is shown for both IPF and non-IPF studies: it is very close in value and statistically highly significant for both groups.

The impetus to perform this meta-analysis was a combination of recent studies on non-IPF and the recognition of the absence of effective therapy in progressive fibrosis in these conditions. A review by Athol Wells in 2018 summarised the data for the following propositions: that there is IPF-like disease progression in subgroups of patients with non-IPF progressive disease; and there may be a common pathway, that is, common pathogenetic mechanisms leading to fibrosis [20]. The authors of the current review recognise that the two anti-fibrotic agents grouped together in this analysis have different mechanisms of action. We are addressing the hypothesis that, since they have a similar efficacy in treating IPF, then a similar pooled degree of efficacy will be seen when treating progressive fibrosis in non-IPF, provided the underlying pathogenetic mechanisms leading to fibrosis in non-IPF are sufficiently similar to those in IPF. These ideas have been expressed elsewhere [21]. They are supported by a re-analysis of the INBUILD study, where subgroups of subjects with hypersensitivity pneumonitis, autoimmune ILDs, idiopathic non-specific interstitial pneumonia, unclassifiable idiopathic interstitial pneumonia, and other ILDs were analysed separately, and the protective effect of anti-fibrotic therapy was similar regardless of the subgroup [22]. Our meta-analysis on changes in lung function supports the above hypothesis. The data on mortality are insufficient to allow any firm conclusions, although we know that changes in lung function provide the best predictor for mortality in these conditions [23].

One important aspect of any meta-analysis is assessment of heterogeneity. The assumption when using a meta-analysis to compare two groups, in this case IPF and non-IPF, is that we can reliably measure heterogeneity in the data: that is, the variation between study effect sizes attributable to the true distribution of effect sizes with the sampling error removed. In our study, addressing the standardised effect size of FVC decline for all studies, no statistically significant evidence of heterogeneity was found: the Q value was 13.9, where the expected value was 12, and the excess variation above expected was not statistically significant. The prediction interval calculated assuming heterogeneity was narrow; from which we can say that if we have failed to detect heterogeneity, it is of a modest degree. It can be supposed that in this meta-analysis, heterogeneity for the standardised change in FVC has been estimated with sufficient precision to draw conclusions: the number of studies was adequate, and greater than 10, which is sometimes given as a ballpark figure for adequate estimation of heterogeneity; all the studies were RCT’s with narrow and similar inclusion criteria (other than for the disease state); and nine of the studies were of good size with greater than 100 patients in the treatment arm. This is our justification for including only RCT’s: incorporation of other types of study such as cohorts can introduce bias by well-known mechanisms and hence heterogeneity that arises from the type of study rather than from the condition being studied [24]. The data support the existence of a clinically important benefit of antifibrotic therapy in both IPF and non-IPF, even if that effect is not identical.

Even in a RCT of ILD, the outcomes can be biased if differences in treatment within the study period, particularly of exacerbations, have unintended consequences. The well-known PANTHER-IPF trial in IPF patients published in 2012 was terminated after about 50% of the planned data had been collected because an interim analysis showed increased mortality compared with placebo in the group treated with a combination of prednisolone, azathioprine and NAC [25]. The figures were 8 deaths of 77 patients on the combination therapy versus 1 death in 78 patients on placebo, over the course of 60 weeks. The interim analysis performed showed no difference in the rate of FVC decline between treatment and placebo groups. It is reasonable, given the subsequent data on NAC, to attribute the excess mortality in IPF to the combination of prednisolone and azathioprine. That leaves open the possibility that either or both of these drugs may have an adverse effect in IPF if given individually as opposed to in combination. A recent study strongly questions the wisdom of an intense anti-inflammatory treatment regimen incorporating methyl prednisolone and cyclophosphamide in the treatment of severe exacerbations of IPF, showing an increase in mortality where cyclophosphamide was added [26]. It was therefore of interest to examine the extent of use of anti-inflammatory medication in the studies included in this review. Of the studies of nintedanib in IPF, about 21% of the study population was on Prednisolone up to 15 mg daily, with minimal use overall in the studies of pirfenidone in IPF. It is unlikely any significant bias was introduced by anti-inflammatory therapy in the studies included for IPF.

Anti-inflammatory therapy was widely used in the non-IPF studies, with a frequency between 5 and 63% of the study populations, and varied markedly between studies. Although anti-inflammatory therapy is frequently administered in non-IPF lung disease, the evidence for its efficacy (or harm) in progressive non-IPF is variable [27–29]. The Distler study shows a benefit of nintedanib on spirometry that was independent of benefit from Mycophenolate, but no separate analysis of effect of anti-inflammatory therapy on efficacy was made in the other studies included, so no conclusion on this can be drawn.

Limitations apply to this meta-analysis. The studies for non-IPF contain more patients on nintedanib than pirfenidone (494 versus 153), so conclusions for non-IPF can be more confidently applied to nintedanib than pirfenidone, although no significant heterogeneity in outcomes was shown. For FVC data, the method of analysis of data led to extracted data being in both an absolute metric (mls) or relative to predicted values, necessitating the use of standardised differences. The use of ranked data analyses is justifiable statistically, but does not give the reader a clear handle for the change that has been demonstrated. Different approaches to imputation of data also complicate interpretation of the scale of effects, although a review of data imputation techniques for the ASCEND trial showed that the outcomes are robust in relative terms, that is to say, the magnitude of the absolute changes in FVC was significantly affected, but the effect of the anti-fibrotic therapy remained about a 50% inhibition of the control response [30]. This offers further justification for the use of a standardised difference in meta-analysis, since this is unaffected by the scale of the outcome. For mortality data, in an ideal world, one would have hazard ratios from the included studies, which can readily be pooled with meta-analysis. We had to be content with comparison of deaths as events in a binomial analysis, which is a close approximation where deaths remain a small proportion of the total subjects over the study period.

We believe that this meta-analysis is the first to attempt to pool data from RCT’s on both commonly used anti-fibrotic drugs in both IPF and non-IPF. Several cohort studies, some looking at both pirfenidone and nintedanib in IPF have been published, including a recent large retrospective cohort analysis which excluded systemic sclerosis associated ILD and progressive fibrosing ILD [31]. We have discussed above the problems with cohort studies and the reasons for selecting RCT’s in this analysis. We chose to focus solely on the parameters of spirometric changes and all-cause mortality as being clinically relevant and the most robust measures that could be extracted from multiple studies. Other parameters such as time to exacerbation and quality of life are clearly important, but they are not reported in all of the studies included in this analysis. In addition, definitions of exacerbations vary and the use of different instruments to assess quality of life makes comparisons difficult.

## Conclusions

For changes in FVC, anti-fibrotic therapy offers protection against the rate of decline, similar between the two anti-fibrotic agents currently in clinical use, with no obvious difference in efficacy whether the underlying condition was IPF or non-IPF with progressive fibrosis. Larger controlled studies in non-IPF are required to be confident about any general effect on mortality by anti-fibrotic therapy.

## Data Availability

Not applicable.

## Abbreviations

CI: Confidence interval (95% unless otherwise stated)
FVC: Forced vital capacity
IPF: Idiopathic pulmonary fibrosis
Non-IPF: Pulmonary disease with progressive fibrosis not classified as IPF
RCT: Randomised controlled trial
